# Disabling complement regulatory activities of vaccinia virus complement control protein reduces vaccinia virus pathogenicity

**DOI:** 10.1016/j.vaccine.2011.07.062

**Published:** 2011-10-06

**Authors:** John Bernet, Muzammil Ahmad, Jayati Mullick, Yogesh Panse, Akhilesh K. Singh, Pradeep B. Parab, Arvind Sahu

**Affiliations:** National Centre for Cell Science, Pune University Campus, Ganeshkhind, Pune 411007, India

**Keywords:** Smallpox vaccine, Vaccinia virus, VCP, Complement, Immune evasion

## Abstract

Poxviruses encode a repertoire of immunomodulatory proteins to thwart the host immune system. One among this array is a homolog of the host complement regulatory proteins that is conserved in various poxviruses including vaccinia (VACV) and variola. The vaccinia virus complement control protein (VCP), which inhibits complement by decaying the classical pathway C3-convertase (decay-accelerating activity), and by supporting inactivation of C3b and C4b by serine protease factor I (cofactor activity), was shown to play a role in viral pathogenesis. However, the role its individual complement regulatory activities impart in pathogenesis, have not yet been elucidated. Here, we have generated monoclonal antibodies (mAbs) that block the VCP functions and utilized them to evaluate the relative contribution of complement regulatory activities of VCP in viral pathogenesis by employing a rabbit intradermal model for VACV infection. Targeting VCP by mAbs that inhibited the decay-accelerating activity as well as cofactor activity of VCP or primarily the cofactor activity of VCP, by injecting them at the site of infection, significantly reduced VACV lesion size. This reduction however was not pronounced when VCP was targeted by a mAb that inhibited only the decay-accelerating activity. Further, the reduction in lesion size by mAbs was reversed when host complement was depleted by injecting cobra venom factor. Thus, our results suggest that targeting VCP by antibodies reduces VACV pathogenicity and that principally the cofactor activity of VCP appears to contribute to the virulence.

## 1 Introduction

The first barriers that microorganisms including viruses must breach for being successful pathogens are imposed by the innate immune system of which the complement system constitutes a major arm [1–4]. The complement system comprises of an intricate group of both soluble and cell-associated proteins activated through three major pathways, the classical, alternative and lectin pathways. Complement activation results in the generation of active components, including C3b and C4b, which aid in the assembly of enzymes called as C3/C5-convertases that facilitate downstream cleavage and formation of the membrane attack complex (MAC) capable of lysing pathogens. Additionally, the activation products C3a and C5a show anaphylatoxic and chemotactic properties [5] and also play a role in T cell activation [6], and surface bound complement components derived from C3 interact with specific immune receptors, thus acting as a connecting link with the adaptive immune system [7]. Hence, the complement system exerts assault on pathogens directly by lysis and indirectly by boosting the pathogen-specific immune responses [8].

Variola virus, the etiological agent of smallpox, caused large-scale mortality and morbidity among humans before its successful eradication. Even though smallpox has been eradicated there are two major concerns related to poxviruses, one of which is the possibility of usage of variola as a bioterrorism agent and the other being cross-species related infections, e.g., monkeypox and cowpox virus infection of humans [9–11], requiring further understanding of the pathogenesis of this complex group of viruses.

Complement activation either through the alternative pathway or through the classical pathway plays a pivotal role in the neutralization of poxviruses. Vaccinia virus (VACV), the prototypic poxvirus, has two major forms: the extracellular enveloped (EV) and the intracellular mature virus (MV). Among these, the EV form is more resistant to neutralization by antibodies, but this is reversed in the presence of complement [12]. This is further highlighted by the observation that both in vitro and in vivo neutralization of the EV form could be achieved with antibodies targeted against B5R, an EV form-specific protein, in the presence of complement [13]. These studies besides emphasizing the role of antigen specific antibodies also identify the pivotal role complement plays in targeting and neutralizing poxviruses.

Viruses override the complement system by developing various mechanisms to mask themselves against the host's complement assault [14–17]. Poxviruses in particular, have been shown to encode mimics of human regulator of complement activation (RCA) proteins to target complement, besides the additional strategy of recruitment of human RCAs [18–21]. Vaccinia and variola viruses, the two important members of the genus *Orthopoxvirus* [22,23], encode soluble RCA homologs named vaccinia virus complement control protein (VCP) and smallpox inhibitor of complement enzymes (SPICE), respectively [24,25]. Both effectively inhibit complement, with SPICE being more human specific than VCP [25,26]. Other members of the pox family, like cowpox virus, monkeypox virus and ectromelia, also encode functional RCA mimics with marked identity among the homologs, except monkeypox virus strains, which have been shown to either lack or have a truncated form of the homolog [20,27–31].

VCP is entirely formed by four complement control protein (CCP) domains separated by short linkers, which is a characteristic of the RCA proteins [32–34] and exists either as a secreted or a cell associated form [24,35]. Functional studies revealed that it inhibits the complement-mediated neutralization of both the infectious forms of VACV i.e., MV as well as EV [36,37]. Notably, VCP has been shown to be involved in modulating the humoral and T cell mediated responses to VACV infection [38]. Detailed studies on the mechanism of complement inactivation showed that it binds to complement proteins C3b and C4b [39,40] and inhibits complement by targeting C3-convertases, by supporting factor I mediated inactivation of C3b and C4b (cofactor activity) [39,41] and by accelerating irreversible dissociation of the classical pathway C3-convertase (decay-accelerating activity) [39,42]. Structure–function analysis using monoclonal antibodies (mAbs), chimeric proteins and deletion mutagenesis indicated that all the domains contribute to C3b and C4b binding and functional activities [42–45]. Further, our recent domain swapping study with human complement regulators revealed that of the four domains, domain 1 imparts decay of the protease subunit from the C3-convertase and domains 2 and 3 recruit factor I for imparting proteolytic inactivation of C3b and C4b [46].

The importance of VCP in VACV pathogenesis was addressed initially in a study using rabbit and guinea pig intradermal models where it was shown that VACV devoid of VCP produced comparatively smaller lesions than the wild type virus [36]. More recently, similar experiments were also performed using mice ear pinnae model in complement sufficient and C3^−/−^ mice [38]. Though these studies clearly demonstrated the importance of VCP in pathogenesis, the significance of complement inhibition by VCP as a contributing factor in the pathogenesis based on the individual known functions is still not clear. In the present study, we have generated a panel of mAbs against VCP and used them to dissect the regions on VCP that play a role in the complement regulatory activity. We then asked whether VCP could serve as a virulence determinant by targeting complement and if so, which complement regulatory activity of VCP plays a predominant role in virulence in vivo.

## 2 Materials and methods

### 2.1 Virus and proteins

The vaccinia virus strain Western Reserve (VACV-WR) was a kind gift from Dr. Sekhar Chakrabarti (National Institute of Cholera & Enteric Diseases, Kolkata, India). The virus was cultured by infecting African green monkey kidney cells (CV-1) followed by purification on a sucrose gradient. Recombinant VCP (rVCP) and its truncation mutants (CCP 1–3, CCP 2–4, CCP 1–2, CCP 2–3 and CCP 3–4) were expressed and purified as previously described [42]. The complement proteins C1, C2, C4, C4b and factor I were purchased from Calbiochem (La Jolla, CA), and complement proteins factor H, C3 [42] and C3b [41] were purified as described earlier. Human soluble CR1 (sCR1) was a generous gift from Dr. Henry Marsh (AVANT Immunotherapeutics, Inc., Needham, MA). Cobra venom factor (CVF) was purified from *Naja naja kaouthia* venom as described earlier with minor modifications [47]. In brief, the lyophilized venom was dissolved in 20 mM sodium phosphate buffer, pH 7.4, loaded onto Source Q column (12 cm × 9.5 cm, GE Healthcare Bio-Sciences) in the same buffer and eluted with a linear salt gradient to 1 M NaCl. Fractions containing CVF as judged by SDS-PAGE were then purified using Superose 12 column (two columns linked in a series; GE Healthcare Bio-Sciences) followed by Mono Q column. SDS-PAGE analysis showed purity of >95%. Its functionality was verified by its ability to form the stable C3-convertase [47].

### 2.2 Monoclonal antibodies (mAbs)

The VCP specific mAbs were generated by immunizing 5–6 week old BALB/c mice with the rVCP. In brief, mice were immunized with 20 μg of rVCP in Freund's complete adjuvant, followed by two boosts 15 days post prime at weekly intervals with the same dose, but in Freund's incomplete adjuvant. Following immunization, spleen was removed and the spleen cells were fused in-house with myeloma cells as per established protocols [48,49]. The clones from the fusion were screened by ELISA and subcloned to isolate the individual clones. Antibody isotyping was performed by an ELISA-based hybridoma isotyping kit (BD Biosciences, San Diego, CA, USA). The IgG mAbs were purified by capryllic acid precipitation method or by Hi-Trap affinity protein G column (GE Healthcare Bio-Sciences, Sweden). Homogeneity of mAbs was assured by SDS-PAGE analysis.

### 2.3 Rabbit infections

VACV pathogenicity studies were performed in rabbits using skin lesion model [36]. In brief, 10^4^ pfu of VACV-WR strain in sterile PBS in a total volume of 100 μl were injected intradermally with or without the mAb on the shaved backs of two New Zealand White rabbits (age 6–7 months) in duplicate and lesions formed (scabs) were measured after every 24 h using calipers. The mean of four measurements was used for graphical representation of individual time point per site. To study the role of complement during infection, similar experiments were also performed in two additional rabbits depleted of complement by administering 100 U/kg of cobra venom factor. All the results were grouped and statistically evaluated by performing Mann–Whitney Rank Sum test (SigmaStat). The experimental protocol was approved by the Institute's Animal Care and Use Committee.

### 2.4 Binding of mAbs to VCP and VCP truncation mutants

The ELISA plates were coated overnight at 4 °C with rVCP or VCP mutants (CCP 1–3, CCP 2–4, CCP 1–2, CCP 2–3, CCP 3–4; 200 ng/well), blocked by adding 5% milk and incubated with mAbs (1 μg/well) for 1 h at room temperature. Binding was probed by adding 1:2000 diluted anti-mouse HRP conjugate (Biorad, Hercules, CA) and detected with 2,2′-Azino-bis (3-ethylbenzthiazoline) 6-sulfonic acid (ABTS) (Roche, Mannheim, Germany) at 414 nm.

### 2.5 Inhibition of factor I cofactor activity of VCP by mAbs

Inhibition of factor I cofactor activity of VCP by mAbs was determined as described below. rVCP (0.5 μg) was mixed with 3 μg of mAb and incubated for 15 min at 37 °C. Thereafter, 3 μg of C3b or C4b and 0.1 μg of factor I was added to the reaction mixture and the volume was adjusted to 20 μl using PBS. It was then further incubated at 37 °C for 2 h. The reaction was stopped by adding SDS-PAGE sample buffer containing DTT and C3b/C4b cleavages were analyzed on a 10% SDS-PAGE gel [40].

### 2.6 Inhibition of classical pathway decay-accelerating activity of VCP by mAbs

Inhibition of the classical pathway decay-accelerating activity of VCP by mAbs was determined by utilizing a hemolytic assay [42,50]. Briefly, 10 μl of EAC142 (classical pathway C3-convertase; 1 × 10^9^ cells/ml) was mixed with 0.2 μM of rVCP and 1 μM of mAb in a total volume of 25 μl and incubated for 5 min at 22 °C. The remaining C3-convertase activity was determined by measuring hemolysis after incubating the reaction mixture for 30 min at 37 °C with 1:100 diluted guinea pig serum containing 40 mM EDTA. Hemolysis was measured at 405 nm.

### 2.7 Surface plasmon resonance measurements

The kinetics of binding of the mAbs to VCP was determined on the SPR-based biosensor BIACORE 2000 (Biacore AB, Uppsala, Sweden). All the experiments were performed in PBS-T (10 mM sodium phosphate, 145 mM NaCl, pH 7.4 containing 0.05% Tween 20) at 25 °C. About 2600 RUs of each of the mAbs was immobilized on test flow cells (Fc-2, Fc-3 or Fc-4) of a CM5 chip using amine-coupling chemistry and non-immobilized flow cell (Fc-1) served as the control flow cell [40]. Various concentrations of rVCP were then flown over the chip at 30 μl/min for 120 s and dissociation was followed for an additional 180 s. The chip was regenerated by injecting brief pulses of 0.2 M sodium carbonate, pH 9.5. Data obtained for the control flow cell were subtracted from those obtained for test flow cell and evaluated using BIAevaluation software version 4.1 using global fitting.

### 2.8 Measurement of half-life of mAbs in rabbits

The half-life of two of the VCP neutralizing mAbs (NCCS 67.5 and 67.9) in rabbits was assessed by radiolabeling the antibodies with ^131^I. One hundred microliters of the labeled mAbs at a dose of 100–200 μCi (∼65–100 μg) were injected intradermally on backs of New Zealand White rabbits and imaged using an ELGEMS “Millennium MPS” gamma ray camera (GE, USA) at the Department of Veterinary Medicine and Veterinary Nuclear Medicine Center (Mumbai, India). A maximum of 250 kilocounts was set for acquiring images and a medium energy collimator was used to capture emerging radiations. The images were acquired at various time points and analyzed using GENIE acquisition user interface software (GE, USA). The first image acquired immediately after the injection was considered as zero time point. The data obtained were normalized by considering the counts obtained at the zero time point as 100%.

## 3 Results

### 3.1 Anti-VCP monoclonal antibody generation and affinity measurements

To re-examine the VCP domains responsible for complement modulation and to understand the in vivo relevance of these complement regulatory functions in VACV pathogenesis, we raised a panel of mAbs against VCP by immunizing BALB/c mice followed by fusion, and cloning and subcloning of the candidate hybrids. The monoclonal antibodies thus generated largely belonged to IgG1κ isotype. Four antibodies, namely NCCS 67.5, NCCS 67.9, NCCS 67.11 and NCCS 67.13, all belonging to IgG1κ isotype, were chosen for further characterization as they differentially inhibited the functional activities of VCP (see below). Of the four, mAb 67.5 uniquely displayed two distinct light chains, which could be a result of difference in their glycosylation states [51]. The light chains however did not show any binding to Con A suggesting that the difference in their molecular weights was not a result of difference in the presence of Con A binding carbohydrates (Supplementary Fig. 1). Although unusual, the clonal origin of an antibody containing two separate light chains has been reported earlier [52,53]. Thus, it seems that mAb 67.5 may belong to such a category.

**Fig. 1:**
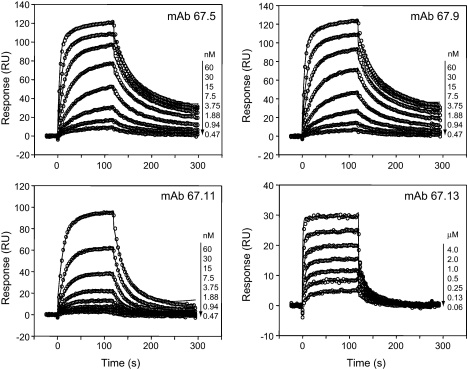
Surface plasmon resonance analysis of binding of anti-VCP monoclonal antibodies to VCP. Sensogram overlay plots of the binding of anti-VCP mAbs 67.5, 67.9, 67.11 and 67.13 to VCP. The anti-VCP mAbs were immobilized on the CM-5 sensor chips (Biacore AB) using amine-coupling chemistry and various concentrations of VCP (indicated on right hand side of each panel) were flown over the chip in PBS containing 0.05% Tween 20 to measure binding. The solid black lines represent the best fit data to 1:1 Langmuir binding model (A + B ↔ AB; BIAevaluation 4.1).

All the four antibodies bound to VCP in a direct ELISA (data not shown). Since surface plasmon resonance (SPR) offers more quantitative data on biomolecule interactions, we utilized this method to measure the affinities of these antibodies. In the SPR setup, the antibodies were immobilized onto the chip and rVCP was flowed over it to measure binding. All the antibodies bound to VCP in a dose dependent manner (Fig. 1). The equilibrium dissociation constant (*K*_D_) of mAbs 67.5 and 67.9 (5.35 × 10^−9^ M and 6.6 × 10^−9^ M) was lower compared to those of 67.11 (4.64 × 10^−8^ M) and 67.13 (2.32 × 10^−7^ M), respectively (Table 1). Interestingly, in a dot blot assay, these mAbs bound to VCP only under non-reducing conditions (data not shown) indicating that these antibodies recognize the conformational epitopes on VCP.

### 3.2 Mapping of VCP domains recognized by anti-VCP monoclonal antibodies

**Fig. 2:**
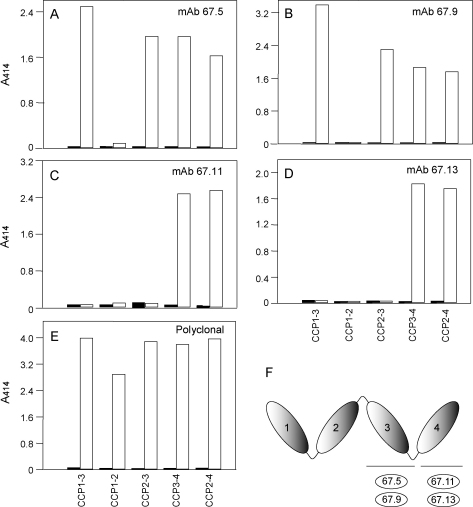
Mapping of VCP domains recognized by anti-VCP monoclonal antibodies. Identification of VCP domains recognized by the mAb was performed by examining the binding of the mAbs to various VCP truncation mutants by ELISA. The filled bars represent binding of the mAbs to the control (BSA), while the empty bars represent binding of the mAbs to the indicated VCP truncation mutant. Panels (A, B, C, and D) depict binding of mAbs 67.5, 67.9, 67.11 and 67.13, respectively, and panel (E) represent binding of VCP to rabbit polyclonal antibody (control). The schematic summary of VCP domains recognized by the various mAbs is presented in panel (F).

To identify the VCP domains to which these mAbs bind we performed an indirect ELISA using various truncation mutants of VCP (CCP 1–3, CCP 2–4, CCP 1–2, CCP 2–3 and CCP 3–4) that were expressed earlier in our laboratory using the *Pichia* expression system [42]. These expressed mutants were designed in such a way that they started with the first Cys of the domain of interest and ended with the last residue of the inter-domain linker. Thus, this design kept the entire linker region at the C-terminal side of each of the mutants. The mAbs 67.5 and 67.9 reacted with CCP 1–3, CCP 2–4, CCP 2–3 and CCP 3–4 mutants (Fig. 2A and B) indicating that they recognize either domain 3 or the linker between domains 3 and 4 (Fig. 2F). The antibodies 67.11 and 67.13 on the other hand reacted only with truncation mutants CCP 2–4 and CCP 3–4 (Fig. 2C and D). Since the latter two antibodies did not show binding to CCP 1–3, CCP 1–2 or CCP 2–3 it indicates that the binding epitopes for these antibodies lie on CCP domain 4 (Fig. 2F).

### 3.3 Inhibition of C3b and C4b cofactor activities of VCP by anti-VCP monoclonal antibodies

**Fig. 3:**
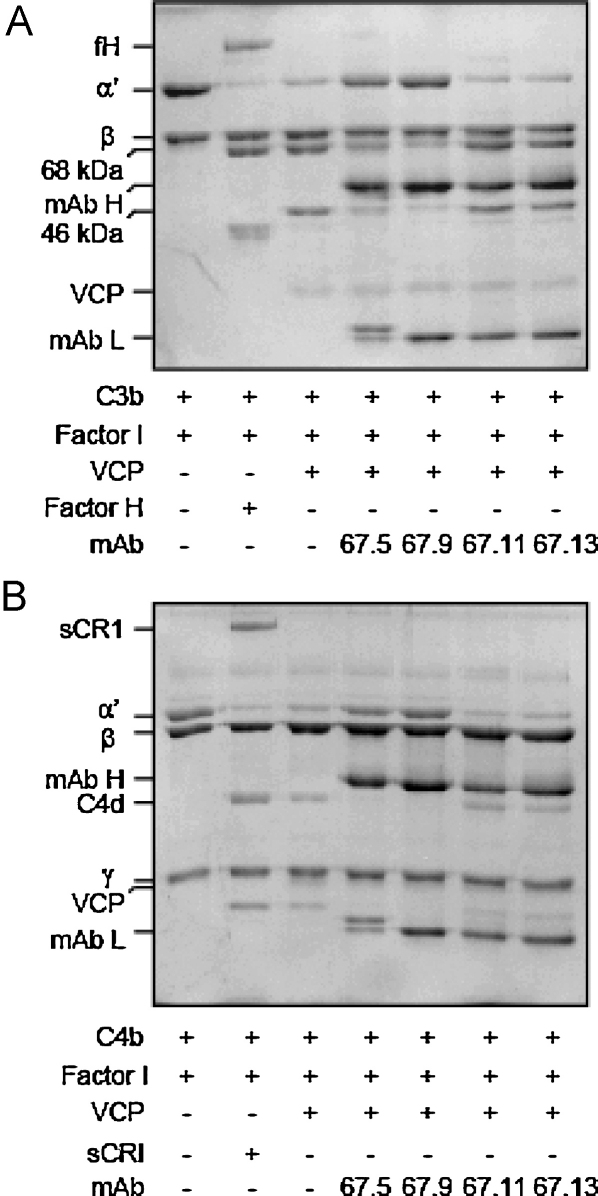
Inhibition of factor I cofactor activity of VCP for C3b and C4b by anti-VCP monoclonal antibodies. (A) Inhibition of C3b cofactor activity. C3b (3 μg) was incubated with VCP (0.5 μg), factor H (1 μg; fH; control) or VCP (0.5 μg) pre-incubated with each of the mAbs (where indicated) and factor I (100 ng) at 37 °C for 2 h. The reactions were stopped by adding SDS-PAGE sample buffer containing DTT and ran on SDS-PAGE gels. (B) Inhibition of C4b cofactor activity. C4b (3 μg) was incubated with VCP (0.5 μg), soluble complement receptor 1 (1 μg; sCR1; control), or VCP (0.5 μg) pre-incubated with each of the mAbs (where indicated) and factor I (100 ng) at 37 °C for 2 h. The reactions were stopped by adding SDS-PAGE sample buffer containing DTT and ran on SDS-PAGE gels. The gels were stained with Coomassie blue to visualize inhibition of α′-chain cleavage, which indicates inhibition of C3b or C4b cofactor activity.

One of the functions of VCP is to serve as a cofactor for the complement specific serine protease factor I to mediate the inactivation of C3b (composed of α′ and β chains) and C4b (composed of α′, β and γ chains), the non-catalytic subunits of C3-convertases, which are the key enzymes in activation of the complement cascades. This function results in the cleavage of the α′-chains of C3b and C4b leading to the generation of their inactivated forms (iC3b or C4c and C4d) which can no longer participate in the formation of C3-convertases. As expected, incubation of rVCP or human factor H (control) with C3b and factor I resulted in cleavage of α′-chain of C3b (Fig. 3A). Similarly, incubation of rVCP or human sCR1 (control) with C4b and factor I resulted in cleavage of α′-chain of C4b (Fig. 3B). When mAbs were analyzed for their ability to inhibit the C3b cofactor activity of VCP, only 67.5 and 67.9 showed inhibition; neither 67.11 nor 67.13 could inhibit this activity (Fig. 3A). Essentially similar results were obtained for inhibition of C4b cofactor activity by the monoclonal antibodies. Only 67.5 and 67.9 showed inhibition, while 67.11 and 67.13 failed to inhibit the C4b cofactor activity (Fig. 3B). These data therefore revealed that CCP domain 3 and/or linker between CCPs 3 and 4 of VCP play an essential role in imparting the cofactor activities.

### 3.4 Inhibition of decay-accelerating activity of VCP by anti-VCP monoclonal antibodies

**Fig. 4:**
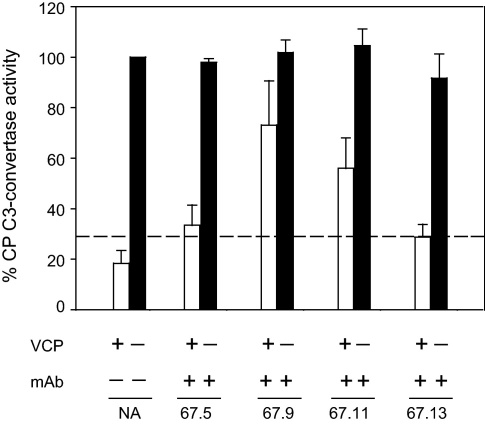
Inhibition of classical pathway decay-accelerating activity of VCP by anti-VCP monoclonal antibodies. The classical pathway (CP) C3-convertase was formed on sheep erythrocytes and allowed to decay in the presence of VCP (0.2 μM) or VCP (0.2 μM) and antibody (1 μM). The remaining C3-convertase activity was detected by adding EDTA-sera (a source of C3-C9). The percentage of CP C3-convertase activity was normalized by considering the percentage of lysis in the absence of VCP as 100%. The dotted line was used as a cut off for determining the inhibition of VCP mediated decay of C3-convertase by mAb. NA indicates controls where no antibody was added.

Besides acting as a cofactor for C3b and C4b inactivation, VCP is also an efficient decay accelerator of the classical/lectin pathway C3-convertase C4b,2a. Thus, to examine the effect of mAbs on VCP-mediated decay of the convertase, we utilized a hemolytic assay. In this assay, C4b,2a was formed on antibody sensitized sheep erythrocytes using purified complement components and then the enzyme was allowed to decay in the presence of rVCP or rVCP pre-incubated with each of the mAbs. The activity of the remaining enzyme was assayed by adding EDTA-sera (a source of C3-C9) and measuring hemolysis. Interestingly, the antibodies that inhibited the C3b and C4b cofactor activities (67.5 and 67.9) also inhibited the decay-accelerating activity of VCP, albeit with 67.5 having much less effect compared to 67.9. Among the remaining two antibodies 67.11 and 67.13, which bound to CCP 4 domain, only the former had moderate inhibitory activity while the latter did not inhibit the decay activity. The C3-convertase decay inhibition efficiency of the monoclonals followed the order 67.9 ≈ 67.11 > 67.5 with 67.13 having negligible inhibitory potential (Fig. 4).

### 3.5 Blocking VCP function using monoclonal antibodies reduces VACV pathogenicity in rabbits

Since mAbs differentially inhibited the VCP functions it was intriguing to know if blocking VCP function in vivo with these mAbs would translate into differences in viral pathogenesis.

**Fig. 5:**
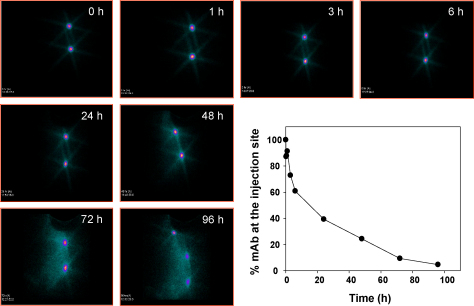
Half-life of anti-VCP mAb in rabbit. Anti-VCP mAb 67.5 was labeled with ^131^I and injected intradermally at two sites in the back of New Zealand White rabbit (NZW) followed by Gamma-ray imaging. The emitted radiation from the sedated animal was imaged with an ELGEMS Millennium MPS gamma ray camera (GE, USA) using a medium energy collimator. The figure shows images of the residence of the labeled mAb at the indicated time points and the graph represents percentage of counts at the indicated time period. The data was normalized by setting 100% equal to the total counts present at 0 time point.

For in vivo disabling of VCP using mAbs, a prerequisite is that they should be retained at the site of injection until VCP is secreted by the infected cells. To verify this, we determined their half-life. The mAbs (67.5 and 67.9) were labeled with ^131^I, injected intradermally on either flanks of New Zealand White rabbits and imaging was carried out with a γ-ray camera. The results showed that the labeled antibodies were retained at the site of injection even after 72 h. The half-life was found to be 8 h for both the antibodies (Fig. 5; data not shown for 67.9).

**Fig. 6:**
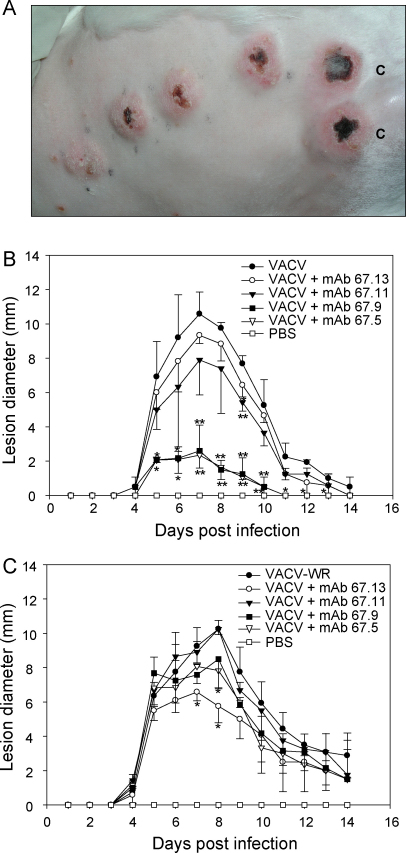
Monoclonal antibody effect in vivo in a rabbit skin lesion VACV-WR challenge model. (A) Skin lesions developed after intradermal injection of 10^4^ pfu of VACV-WR (extreme right lesions marked as C) or 10^4^ pfu of virus mixed with graded concentrations of mAb 67.5 (25 μg, 12.5 μg, 6.3 μg and 3 μg, first four lesions from left). The image was taken 8 days post infection. (B) Skin lesion size after intradermal injection of 10^4^ pfu of VACV-WR alone or in combination with indicated antibodies (25 μg) in rabbits. (C) Skin lesion size after intradermal injection of 10^4^ pfu of VACV-WR alone or mixed with 25 μg of the indicated antibodies in rabbits depleted of complement by injecting cobra venom factor 6 h prior to infection. Data points presented in panels (B and C) represent mean of four replicates performed in two rabbits. Statistical comparisons were performed between mAb treated lesions and untreated lesions. *p < 0.01 and **p < 0.001 (Mann–Whitney Rank Sum test, SigmaStat). Differences are also significant between 67.13 treated lesions and 67.5/67.11 treated lesions between days 5 and 11 (*p* ≤ 0.001).

Next, in order to determine whether disabling of VCP using neutralizing mAb affects VACV pathogenicity, we used a rabbit skin lesion model. In these experiments, VACV-WR was injected intradermally (10^4^ pfu) either alone or in combination with mAbs and the lesion size was measured over a period of time. Initially, the two blocking antibodies (67.5 and 67.9) were titrated with VACV-WR to identify the optimal concentration required for reduction in lesion response. When varying concentrations of 67.5 (Fig. 6A) or 67.9 (data not shown) were mixed with VACV-WR and injected in rabbits they showed a dose dependent inhibition of lesion formation. Maximum decrease in the lesion size was observed at 25 μg mAb concentration. We then performed experiments with all the four mAbs using a fixed concentration (25 μg). There was a significant difference in the lesion size where 67.5 or 67.9 was injected along with VACV-WR (Fig. 6B). Moderate decrease in the lesion development was also observed where 67.11 was injected, but 67.13 showed a negligible effect on the lesion development. These data therefore suggested that in vivo inhibition of complement regulatory activities of VCP by neutralizing mAbs result in reduction in VACV pathogenesis.

### 3.6 Neutralizing monoclonal antibodies do not reduce VACV pathogenicity in complement-depleted rabbits

Although the above results suggested that blocking of complement regulatory activities of VCP by mAbs resulted in neutralization of virus and in turn its pathogenicity, it still did not provide direct evidence of a role of host complement. Consequently, we performed similar experiments in two complement-depleted animals.

Complement depletion in rabbits was achieved by injecting CVF. A bolus of 100 U/kg administered through the ear vein completely depleted complement in rabbits in 4 h and the depleted state was maintained till day 5, after which there was a gradual restoration of complement activity (data not shown). Because it took approximately 4 h to completely deplete complement in rabbits, in these experiments, we injected CVF 6 h prior to the challenge in duplicate with VACV-WR or VACV-WR along with mAb in the back of each rabbit. It is clear from our data that intradermal injection of VACV-WR (10^4^ pfu) with or without mAb (25 μg of each) led to the formation of more similar sized lesions (Fig. 6C). It could therefore be suggested that inhibition of VCP-mediated regulation of host complement by neutralizing antibodies result in neutralization of VACV in a host complement dependent manner.

## 4 Discussion

VCP is one of the most extensively studied pox viral RCA homologs [4,54,55]. It is now clear that it possesses the ability to regulate the complement system in the fluid phase as well as on the surface of the infected cells by binding to heparan sulfate proteoglycans [56] and the viral protein A56 [35]. Further, it has also been established that its deletion causes attenuation of VACV lesion and increase in specific inflammatory responses in mice [36,38]. However, the in vivo role of its complement interacting domains and importance of its various inhibitory activities with relevance to in vivo pathogenesis is still not understood. In the present study, we have raised neutralizing mAbs against VCP, mapped the domains they recognize and utilized them to address the in vivo relevance of different functional activities of VCP in VACV pathogenesis.

Prior to this study, mAbs against VCP have been generated by Isaacs et al. [45] and Liszewski et al. [57]. The former study by Isaacs et al. [45] showed that mAbs directed against CCP domains 2 and 4 inhibit VCPs interaction with its target proteins C3b and C4b and thereby its activity. And later Liszewski et al. [57] demonstrated that mAb that recognizes the linker between CCP domains 1 and 2 inhibit the cofactor as well as decay-accelerating activity of VCP. Although these studies established the importance of CCP domains 2 and 4 and the linker between domains 1 and 2 in VCPs target recognition and functional activities, no attempts were made in these studies to utilize the antibodies to dissect the in vivo importance of complement regulatory activities of VCP in VACV virulence.

In the present study, we have characterized four mAbs of which two (67.5 and 67.9) recognized domain 3 or the linker between domains 3 and 4, and the other two (67.11 and 67.13) recognized domain 4. Of these four antibodies, 67.5, 67.9 and 67.11 inhibited the complement regulatory activities of VCP (Figs. 3 and 4) suggesting that domains 3 and 4 are critical for the VCP function. This however is not surprising as domain mapping employing chimeric mutants, truncation mutants and mAbs indicated that all the four domains of VCP are important for its interaction with C3b and C4b [42–45]. In addition, we now also know that CCP domains 2 and 3 provide a docking surface for factor I and thus are critical for the cofactor activity, and CCP domain 1 is essential for displacement of C2a from the C3-convertase C4b,2a (decay activity) [46]. In light of these data on domain requirements in VCP for its functional activities, it is likely that the mAbs 67.5 and 67.9 exert their effect by inhibiting the interaction of VCP with C3b/C4b and/or factor I and mAb 67.11 exercises its effect by inhibiting the interaction of VCP with C3b/C4b.

The mAbs characterized here displayed differential effect on the cofactor and decay activities of VCP. The mAb 67.5 primarily inhibited the cofactor activity, 67.9 inhibited both the cofactor activity and the decay-accelerating activity, 67.11 inhibited only decay-accelerating activity and 67.13 did not inhibit any of the activities (Figs. 3 and 4). Hence, these were suitable to gain insight into the role of these activities of VCP in VACV pathogenesis. Here we employed the rabbit intradermal model to study the effect of these neutralizing antibodies on VACV pathogenesis [36]. Injection of mAbs 67.5 and 67.9 along with VACV showed significant reduction in the lesion size when compared to the lesions formed by VACV alone or VACV injected with the control antibody (67.13) (Fig. 6A and B), indicating that like deletion of VCP from VACV [38,46], disabling of VCP functions also leads to attenuation of VACV lesions. Interestingly, mAb 67.11 that inhibited only the decay-accelerating activity of VCP had no significant effect on the lesion formation suggesting thereby that the cofactor and not the decay-accelerating activity plays a major role in contributing to virulence (Fig. 6B). Nonetheless, there are a few caveats. The affinity of 67.11 for VCP is about 10-fold less compared to 67.9 (Fig. 1 and Table 1) and therefore it is also likely that the observed difference in lesion formation is due to the variation in the affinities of these two antibodies for VCP. Interpretation of the viral pathogenicity data employing mAb 67.11 is further complicated by the fact that even in the in vitro DAA assay, its effect was a relatively modest one. A closer look at the data however show that mAb 67.5, despite having comparatively poor DAA inhibitory activity than 67.9, is equally efficient in inhibiting the lesion formation. Moreover, both these antibodies display similar affinities for VCP (Fig. 1 and Table 1). Thus, it is likely that cofactor activity is a major contributor to VCP-mediated enhancement of VACV pathogenesis.

Monkeypox virus strains like Central African (Congo basin) strain encodes a complement regulator (ORF D14L), while the West African strain lacks this protein [28]. A comparison of the West African strain versus the Congo basin strain shows the latter to be more virulent than the former [28] suggesting that complement evasion may play a role in poxvirus pathogenesis. Intriguingly, the Congo basin strain encodes a protein (MOPICE) that contains only cofactor activity and lack the decay activity [21]. Thus, these observations also provide support to our proposal that the cofactor activity of VCP could play a major role in the poxvirus pathogenesis.

Although smallpox has been eradicated from the globe, the recent upsurge in terrorism has increased the concern of usage of variola virus as a biological weapon and has resulted in development of new generation vaccines [11]. The important question therefore is should VCP be present in the new generation vaccine vectors? We show here that disabling of complement regulatory activities of VCP by neutralizing mAbs result in reduction of VACV lesion size in rabbits and this reduction is dependent on the presence of host complement (Fig. 6). Although our study does not define the mechanism responsible for this, clearly there are two possibilities: (i) the lack of VCP-mediated host complement regulation could result in complement-mediated neutralization of VACV in the presence of antibodies that are produced against VACV during the infection and (ii) the lack of VCP-mediated complement regulation could enhance the protective immune response against VACV. Both these probabilities have been established by earlier studies. In support of the first possibility, in vitro studies have shown that complement has the ability to neutralize both MV [36,37] and EV [37] in the presence of anti-VACV antibodies. And an in vivo study has demonstrated complement to be protective against poxvirus infection [58]. Similarly, in support of the second alternative, a recent study has elegantly shown that infection of VCP-null VACV in mice results in enhanced T cells at the site of infection, enhanced neutralizing antibody responses and reduced viral titers [38]. Thus, our study along with these studies suggests that deletion of VCP from the new generation vaccine vectors would result in more safe and effective vaccine vectors against smallpox.

## Figures and Tables

**Figure:**
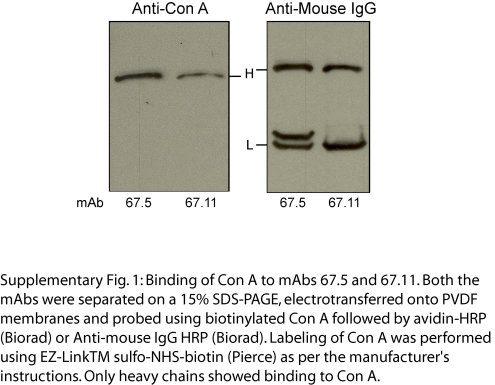


**Table 1:** Kinetic and affinity data for the interactions of monoclonal antibodies with VCP.^a^

Ligand	Analyte	*k*_d_ (1/s)/*k*_a_ (1/M s)	SE (*k*_d_/*k*_a_)	*K*_D_ (M)	*χ*^2^
mAb 67.5	VCP	0.0164/3.07 × 10^6^	9.52 × 10^−5^/1.81 × 10^4^	5.35 × 10^−9^	1.1^b^
mAb 67.9	VCP	0.0171/2.59 × 10^6^	9.96 × 10^−5^/1.51 × 10^4^	6.6 × 10^−9^	1.01^b^
mAb 67.11	VCP	0.034/7.32 × 10^5^	1.73 × 10^−4^/7.32 × 10^5^	4.64 × 10^−8^	0.468^b^
mAb 67.13	VCP	0.0418/1.8 × 10^5^	5.22 × 10^−4^/3.94 × 10^3^	2.32 × 10^−7^	0.218^b^

a *k*_a_, association rate constant; *k*_d_, dissociation rate constant; *K*_D_, equilibrium rate constant; SE; standard error.

b Data were calculated by global fitting to a 1:1 Langmuir binding model (BIAevaluation 4.1).
